# Numerical Investigation of a Compact Dual-Band SIW Filter Operating at 28/38 GHz for 5G Millimeter-Wave Systems

**DOI:** 10.3390/mi17070798

**Published:** 2026-06-29

**Authors:** Khier Benderradji, Boualem Hammache, Idris Messaoudene, Abdallah Hedir, Salem Titouni, Rabia Rebbah, Massinissa Belazzoug, Nadhir Djeffal

**Affiliations:** 1 ETA Laboratory, University Mohamed El Bachir El Ibrahimi, Bordj BouArreridj 34000, Algeria; khier.benderradji@univ-bba.dz (K.B.); boualem_hammache@hotmail.fr (B.H.); salem.titouni@univ-bba.dz (S.T.); rebbahrb@gmail.com (R.R.); m.belazzoug@univ-bba.dz (M.B.); nadhir.djeffal@ummto.dz (N.D.); 2 Laboratoire de Technologies Avancées en Génie Electrique, University of Mouloud MAMMERI, BP 17 RP, Tizi-Ouzou 15000, Algeria; 3 Advanced High Voltage Engineering Centre, School of Engineering, Cardiff University, Queen’s Buildings, The Parade, Cardiff CF24 3AA, UK; 4 Department of Computer Science, National Higher School of Advanced Technologies (ENSTA), Algiers 16031, Algeria; 5 Faculty of Electrical Engineering and Computer Science, University of Mouloud MAMMERI, Tizi Ouzou 15000, Algeria

**Keywords:** dual-band bandpass filter, substrate integrated waveguide (SIW), millimeter-wave, 5G communication systems, Ka-band, compact microwave filter, iris resonator, 28 GHz, 38 GHz, HFSS, CST studio, ADS

## Abstract

With the rapid expansion of 5G millimeter-wave communications, there is a strong demand for compact, low-loss, and high-selectivity filtering components. This paper presents the design and analysis of a compact dual-band substrate integrated waveguide (SIW) bandpass filter operating at 28 GHz and 38 GHz for 5G applications. The proposed structure employs shunt iris-loaded resonators integrated within the SIW cavity to achieve dual-band operation with improved frequency selectivity. The designed filter provides a narrow passband of 1.2 GHz at 28 GHz and a wider passband of 2.6 GHz at 38 GHz, while maintaining a compact footprint of 8.5 mm × 6.2 mm. It is implemented on a Rogers RT/Duroid 5880 substrate ($\varepsilon_{r}$ = 2.2, h = 0.508 mm), ensuring low dielectric loss and stable high-frequency performance. The simulated results demonstrate excellent return loss of 44.2 dB at 28.1 GHz and 52.7 dB at 38.3 GHz, along with a low insertion loss of approximately 0.68 dB, confirming efficient signal transmission. Furthermore, the design is validated using a simulation with ADS of second-order Butterworth equivalent circuit, providing design simplicity and demonstrating the feasibility of practical fabrication. On the other hand, it is well suited for integration into compact 5G front-end modules requiring high performance, miniaturization, and dual-band operation.

## 1. Introduction

**Figure 1:**
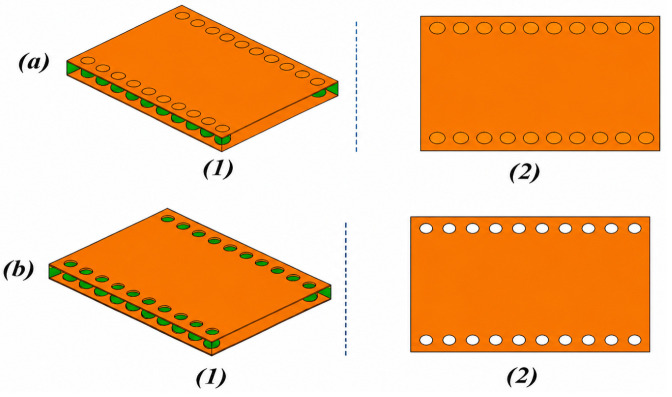
Different modes of metallic via: (**a**) Full via; (**b**) empty via (hole). (1) Isometric view; (2) top/bottom view.

As telecommunications increasingly dominate various sectors, researchers worldwide—especially over the last decade—have focused on developing devices that meet the diverse requirements and standards set by international organizations. These requirements include the allocation of frequency ranges for telecommunication systems, high data speeds, and the rapid processing of information transmitted between transmitters and receivers. To address these demands, components have been developed to operate at very high frequencies, typically in the range of tens of gigahertz, particularly in the millimeter-wave spectrum. Efficient use of the frequency spectrum for data transmission has become essential. This has led to significant advancements in mobile communication technologies, culminating in the introduction of the fifth-generation (5G) networks [1]. Like any wireless transmission system, 5G requires high-frequency ranges to support increased bandwidth. This includes both the sub-6 GHz frequency range and millimeter-wave bands, such as 26, 28, 38, and 60 GHz, enabling data transmission speeds of up to 20 gigabits per second [2,3]. In most electronic systems, essential components like filters play a critical role in separating unwanted signals from those required for specific applications. A bandpass filter allows signals within a specific frequency range to pass through while attenuating signals outside that range. These devices are widely used in the radio frequency (RF) domain and are based on various techniques, including substrate integrated waveguide (SIW) technology, a promising candidate for future advanced telecommunications circuits, where waveguides with a periodic structure can be used as RF devices in various applications [4]. SIW is a modern form of transmission line that has gained popularity due to its efficiency in designing circuits operating in the radio, microwave, and millimeter-wave frequency spectrum. An SIW consists of a thin dielectric substrate, with metallic layers covering both the top and bottom surfaces [5,6]. The substrate contains two parallel rows of metallic via that define the wave propagation area; these via can be empty or full, as illustrated in Figure 1a,b, which represent isometric and top/bottom view, respectively [6].

Researchers have utilized various techniques to realize RF filters. One approach combines single-mode and dual-mode substrate-integrated waveguides (CSMDMSIW) [7]. Another involves designing a wideband grounded coplanar waveguide (GCPW)-to-SIW transition [8]. Some works propose a novel class of compact dual-mode bandpass filters based on half-mode substrate-integrated waveguide (HMSIW) cavities [9], or filters with ultra-wide stopbands using multi-stub-loaded resonators (MSLRs) [10]. Other designs use coupled line resonators (CLR) [11]. Several researchers have proposed ultra-wideband (UWB) bandpass filters with stopband characteristics using a multi-mode resonator (MMR) technique [12,13]. A notable contribution involves a novel class of dual-mode SIW bandpass filters that exploit perturbed SIW rectangular cavities (PSIWRCs) [14]. Additional filters are based on electromagnetic coupling of twin square metamaterial resonators (SRRs) and complementary split-ring resonators (CSRRs) [15]. Other designs include dual-band bandpass filters inspired by a pair of square coupled, interlinked, asymmetric tapered metamaterial resonators [16], when a dual-band filter is a type of electronic filter capable of selectively passing or attenuating signals in two distinct frequency ranges, often with a transition band between them. These filters are crucial in fields such as telecommunications and signal processing, where separating or combining signals at specific frequencies is essential, achieving high selectivity through quadruple-mode, multi-stub-loaded ring resonators (SLRRs) [17]. While some approaches use non-resonant nodes (NRNs) based on single- and dual-mode cavities to form tri-layer stacked SIW structures (TLSSIW using NRN) [18], others utilize multi-mode resonators (MMRs) [19], or even machine learning methods to design compact UWB bandpass filters with notch characteristics using rectangular resonators [20]. Finally, some works propose and design wideband fifth-order bandpass filters using U-slotted substrate-integrated waveguide (USSIW) cavities [21].

In this work, a dual-band passband filter is proposed and designed using substrate integrated waveguide (SIW) technology. The study focuses on the theoretical design and electromagnetic simulation of the filter, while no physical fabrication or experimental manufacturing of the proposed structure has been performed. The proposed filter based on SIW technology operates in the millimeter-wave range, specifically within the Ka band, and resonates at two frequencies allocated for 5G applications: 28 GHz and 38 GHz [5], where the 28 GHz band, corresponding to the 5G NR n257 standard, and the 38 GHz band, which falls within the 5G NR n260 frequency range, are widely recognized as key millimeter-wave bands for 5G communications, offering large bandwidths and supporting high-data-rate wireless services. This compact device demonstrates very good performance, achieving a return loss (RL) of around 44 dB, an insertion loss (IL) of 0.68 dB at 28 GHz, and an RL of around 53 dB, IL of 0.86 dB at the specified frequency of 38 GHz. The simulation of this design and its results are obtained using a high-frequency structure simulator, ANSYS HFSS 15.0, which can be referred to 3D electromagnetic (EM) simulation software to design and simulate high-frequency electronic products, such as antennas, antenna arrays, high-speed interconnects, connectors, IC packages, printed circuit boards, RF or microwave components, and, especially, filters. These results are subsequently validated by the CST Studio Suite 2019 software. Overall, the main contributions of this work are summarized as follows:

- Design and simulation-based of a novel compact substrate-integrated waveguide (SIW) bandpass filter operating at two key 5G frequencies: 28 GHz and 38 GHz, with a small footprint of 10.81 mm × 6.23 mm.
- Demonstration of high performance in the Ka-band with sharp skirt selectivity, low insertion loss (approximately 0.68 dB), and excellent return loss (approximately 43.63 dB at 28.05 GHz and 52.65 dB at 37.96 GHz).
- Integration of a high-performance Rogers RT/Duroid® 5880 substrate, which is manufactured by Rogers Corporation in Chandler, AZ, USA, in order to reduce both physical dimensions and weight, making it suitable for 5G miniaturized components.
- Implementation of a simplified second-order Butterworth equivalent circuit model that facilitates easy fabrication and practical integration into 5G communication systems.

Compared to related works, the proposed approach provides a simplified structure with a reproducible design methodology while maintaining competitive filtering performance in both passbands.

## 2. Theoretical Analysis

**Figure 2:**
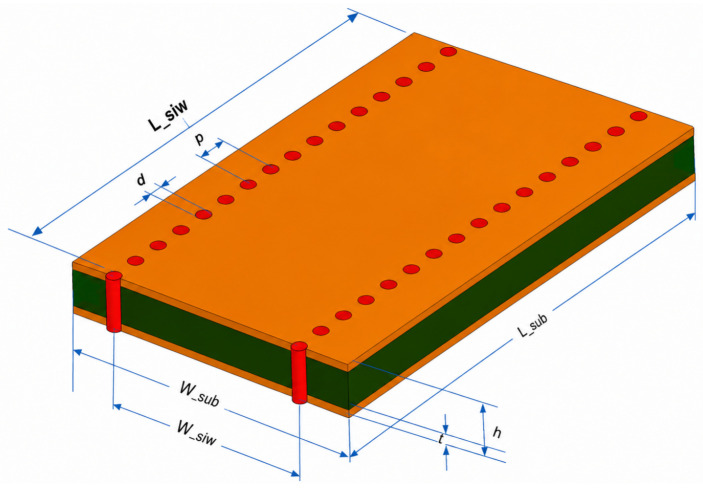
Structure of conventional SIW waveguide.

The substrate integrated waveguide (SIW) filter proposed in this paper is designed using Rogers RT Duroid 5880, a high-frequency laminate material engineered specifically tailored for RF and microwave circuit applications. This substrate is particularly well suited for high-frequency analog and digital circuits operating up to millimeter-wave frequencies. Rogers RT Duroid 5880 is distinguished by its unique combination of low electrical loss and consistent mechanical performance. It features a low-loss tangent (tan *δ* = 0.0009) and an exceptionally low dielectric constant ($\varepsilon_{r}$ = 2.2) at frequencies above 10 GHz. With a thickness of 0.508 mm, it supports stable signal propagation with minimal attenuation, making it ideal for high-performance RF components [13,19]. The dimensions of the substrate are essential for the accurate design of the SIW filter, such as the substrate length ($L_{\mathrm{sub}}$), width ($W_{\mathrm{sub}}$), diameter of the via (*d*), and spacing between adjacent vias (*p*). These dimensions are determined using established formulas [5,22]. These physical parameters, illustrated in Figure 2, influence the filter’s performance, ensuring efficient transmission of electromagnetic waves within the desired frequency range [22,23].

### 2.1. General Case (Single Bandwidth)

The proposed filter was designed to operate at target frequencies of 28 and 38 GHz.

As the SIW is equivalent to a rectangular waveguide where $w_{\mathrm{eff}}\gg h$, the dominant propagation mode is ${\mathrm{TE}}_{m0n}$. The resonant frequency, which depends on the effective width $w_{\mathrm{eff}}$ and effective length $l_{\mathrm{eff}}$ of the waveguide, can be accurately calculated using the following formula [2,4,7,23]:

(1) $$f_{mn}=\frac{c}{2\sqrt{\varepsilon_{r}}}\sqrt{{\left(\frac{m}{w_{\mathrm{eff}}}\right)}^{2}+{\left(\frac{n}{l_{\mathrm{eff}}}\right)}^{2}}$$

Here, the resonance frequency of the dominant mode is $f_{{\mathrm{TE}}_{101}}$. Therefore, the first lower cutoff frequency corresponds to the ${\mathrm{TE}}_{100}$ mode, which can be calculated from Equation (1) by choosing the mode indices (m,n)=(1,0):

(2) $$f_{{\mathrm{TE}}_{100}}=\frac{c}{2\sqrt{\varepsilon_{r}}\cdot w_{\mathrm{eff}}}$$

On the other hand, the upper cutoff frequency, which is $f_{{\mathrm{TE}}_{200}}$, is equal to $2\times f_{{\mathrm{TE}}_{100}}$. In order to obtain a system without leaks, it must choose very suitable values of *d*, *p*, $w_{\mathrm{eff}}$, and $l_{\mathrm{eff}}$. The wavelength at the center resonant frequency of the SIW $\lambda_{o}$ must be found:

(3) $$\lambda_{o}=\frac{c}{f_{{\mathrm{cTE}}_{101}}\times \sqrt{\varepsilon_{r}}}$$

Otherwise, $w_{\mathrm{eff}}$ must be greater than $\frac{\lambda_{o}}{2}$ to obtain a propagation in the system. This is inspired by the following formula for the guide wavelength:

(4) $$\frac{1}{\lambda_{g}^{2}}=\frac{1}{\lambda_{o}^{2}}-\frac{1}{\lambda_{c}^{2}}$$

Therefore, the second part of Equation (4) must be positive, or

$$\frac{1}{\lambda_{o}^{2}}-\frac{1}{\lambda_{c}^{2}}\geq 0$$

where $\lambda_{c}$ represents the wavelength at the cutoff frequency, and is generally equal to twice the effective width of the SIW ($\lambda_{c}\approx 2w_{\mathrm{eff}}$). The width of the SIW ($w_{\mathrm{SIW}}$), represented by the distance between the two walls of the vias, can be calculated through the following relation:

(5) $$w_{\mathrm{SIW}}=w_{\mathrm{eff}}+\frac{d^{2}}{(0.95)p}$$

(6) $$L_{\mathrm{SIW}}=l_{\mathrm{eff}}+\frac{d^{2}}{(0.95)p}$$

Here, *p* and *d* can be found in Equation (7): [5,22,23]

(7) $$\left\{\begin{array}{c} d\leq \frac{\lambda_{c}}{10}\\ p\approx \frac{3}{2}d \end{array}\right.$$

The total width of the SIW ($W_{\mathrm{sub}}$) must be greater than or equal to $w_{\mathrm{eff}}\sqrt{\varepsilon_{r}}$, and the same condition applies to the total length ($L_{\mathrm{sub}}$), which must be bigger than or equal to $l_{\mathrm{eff}}\sqrt{\varepsilon_{r}}$, with the aim of minimizing the losses of the propagation. The insertion of symmetrical shunt spots with diameters equal to $d_{1}$ on the SIW cavity can transform it into a narrow bandpass filter. This device can be fed through a microstrip line that guarantees the transition from the quasi-TEM mode of the microstrip line to the ${\mathrm{TE}}_{101}$ mode of the waveguide.

Generally, an SIW structure without resonators in the middle functions as a traditional waveguide. However, the addition of resonators, such as irises or spots, within the SIW can transform it into a passband filter (PBF) or stopband filter (SBF) [24].

### 2.2. Special Case (Dual Bandwidth)

To design a filter that resonates at two frequencies, each corresponding to a propagation mode, the first step is to start from the first lower cutoff frequency $f_{{\mathrm{TE}}_{100}}$, which is represented in Equation (2), in order to find the effective width of the filter, and then determine the effective length according to the resonance frequency of the dominant mode $f_{{\mathrm{TE}}_{101}}$ according to Equation (1).

**Figure 3:**
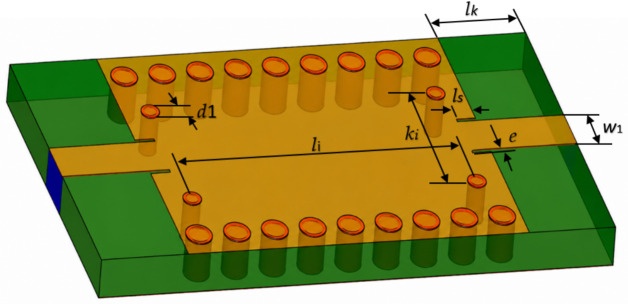
Structure of proposed SIW filter.

Subsequently, the insertion of resonators, positioned vertically apart by a distance $k_{i}$, and horizontally by $l_{i}$, in the form of shunts or spots with a diameter $d_{1}$, as shown in Figure 3, enables resonance at two distinct frequencies. Achieving the desired resonance frequencies simply requires adjusting the dimensions $d_{1}$, $k_{i}$, and $l_{i}$ accordingly [24,25].

### 2.3. Design of the Proposed Filter

As the proposed filter observed in Figure 3 operates at both bandwidths centered around the working frequencies 28 and 38 GHz, the first cutoff frequency must be lower than 28 GHz; and to cover the entire desired bandwidth, $f_{{\mathrm{TE}}_{100}}$ must be at least 24 GHz with $f_{{\mathrm{TE}}_{101}}=28\;\mathrm{GHz}$. For this and according Equations (1) and (2), we obtain

$$\left\{\begin{array}{c} w_{\mathrm{eff}}=4.20\;\mathrm{mm}\\ l_{\mathrm{eff}}=7.07\;\mathrm{mm} \end{array}\right.$$

It is implied that $\lambda_{c}\approx 8.4\;\mathrm{mm}$; therefore, d≤0.84mm according to Equation (7). In addition, $\lambda_{o}$ can be approximately 7.22 mm for the central resonance frequency of 28 GHz, and 5.32 mm for 38 GHz. Thus, $\lambda_{g}=14.12\;\mathrm{mm}$ at 28 GHz and 6.87 mm at 38 GHz according to Equation (4). As the dimensions of the substrate do not change after fixing its length and width to obtain a resonant frequency at 28 GHz for the dominant mode ${\mathrm{TE}}_{101}$, then, in order to obtain another resonant frequency at 38 GHz in the second mode ${\mathrm{TE}}_{201}$, it is necessary to control the appropriate dimensions and positions of the resonators placed inside the filter, especially $l_{i}$, $k_{i}$, and their diameter $d_{1}$.

Based on all the equations mentioned above, all the dimensions or parameters of the proposed filter shown in Figure 3 can be calculated, while those not mentioned in this figure are the same as those shown in Figure 2.

**Figure 4:**
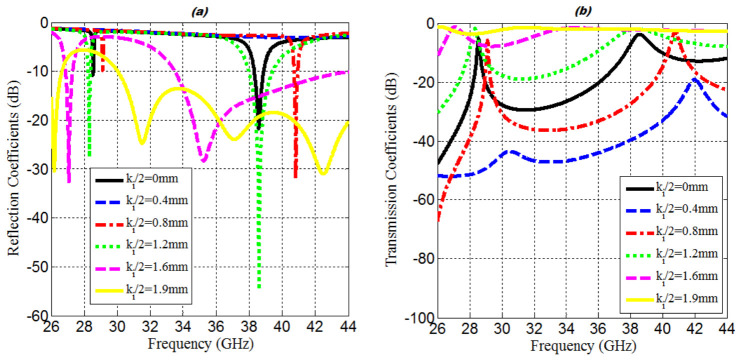
S-parameters for different values of $k_{i}$, (**a**) $S_{11}$, (**b**) $S_{12}$.

**Figure 5:**
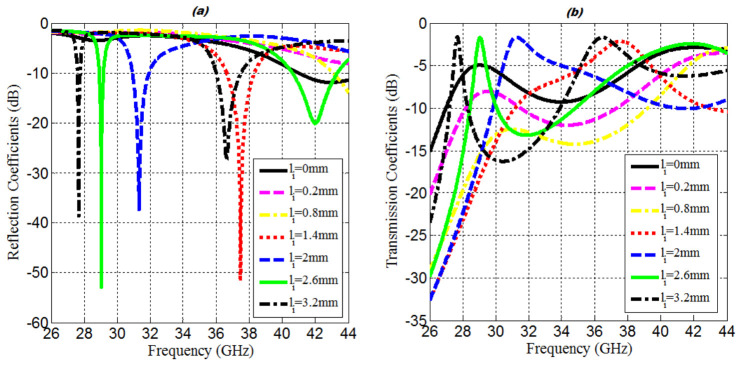
S-parameters for different values of $l_{i}$, (**a**) $S_{11}$, (**b**) $S_{12}$.

**Figure 6:**
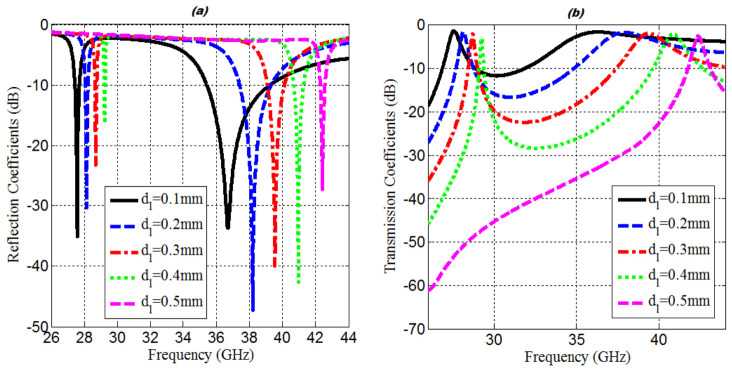
S-parameters for different values of the resonator’s radius $d_{1}$, (**a**) $S_{11}$, (**b**) $S_{12}$.

**Figure 7:**
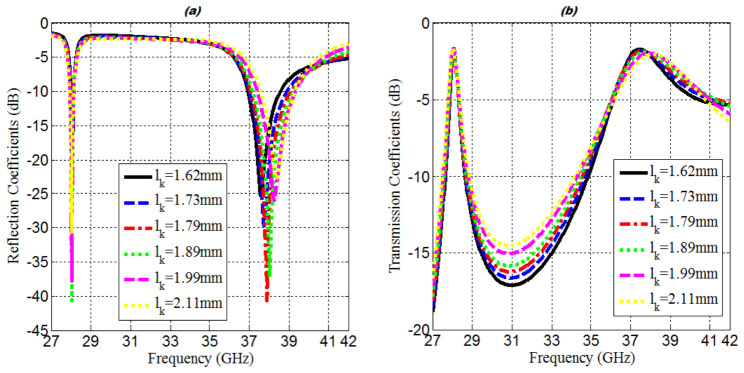
S-parameters for different values of $l_{k}$, (**a**) $S_{11}$, (**b**) $S_{12}$.

The proposed filter is embedded or loaded with resonators in the middle in the form of shunts or cylindrical irises of identical diameters to simplify the calculations; the positioning of these resonators plays a very important role in determining the resonance frequencies [24]. As shown in Figure 4b and Figure 5b, which illustrate the variation of the transmission coefficient $S_{12}$ in dB or IL, the gap between the two passbands decreases or increases depending on the variations of the parameters $l_{i}$ and $k_{i}$. This variation also affects the reflection coefficient $S_{11}$ in dB or RL, which can reach a value of 54.69 dB at a resonance frequency of 38.59 GHz for a $k_{i}$ value of 2.4 mm. On the other hand, an IL value of 52.9 dB is observed at a frequency of approximately 29 GHz for an $l_{i}$ value of 2.6 mm according to what is shown in Part A. In contrast, the impact of the diameters of the resonators on the resonant frequencies and the bandwidth is direct and very clear, as seen in Figure 6 and Figure 7. It is, therefore, clear that the creation of the two bands is ensured by adjusting the distances between the resonators as well as their diameters.

This filter is fed by a microstrip line with a length of $l_{k}$, which can influence its response. This effect is particularly noticeable on the second bandwidth, centered at 38 GHz, as illustrated in Figure 7. It clearly shows the change in the frequency of the second bandwidth based on the increase or decrease in length $l_{k}$. This effect also affects $S_{11}$, where optimal values of return loss (RL) are observed: 35.98 dB at 38.02 GHz and 39.65 dB at 28.05 GHz for a value of $l_{k}=1.89\;\mathrm{mm}$. However, this effect is not significant on the insertion loss (IL) represented by $S_{12}$ in dB, as shown in Figure 7b, except in the second bandwidth around 38 GHz.

## 3. Numerical Results

After determining the appropriate values for the dimensions of the filter, as illustrated in Table 1, we proposed and simulated the design using the EM software, specifically ANSYS HFSS 15.0. We then validated these results using another software, the CST Studio Suite 2019, to confirm their accuracy. It is clearly observed that the curves of the S-parameters in decibels, representing insertion loss (IL) and return loss (RL), are nearly identical, particularly at the resonance frequency of 28 GHz. However, the second passband centered at 38 GHz is noticeably wider in the HFSS simulation, extending up to about 3.6 GHz, compared to the CST simulation, where it is around 2.1 GHz.

**Figure 8:**
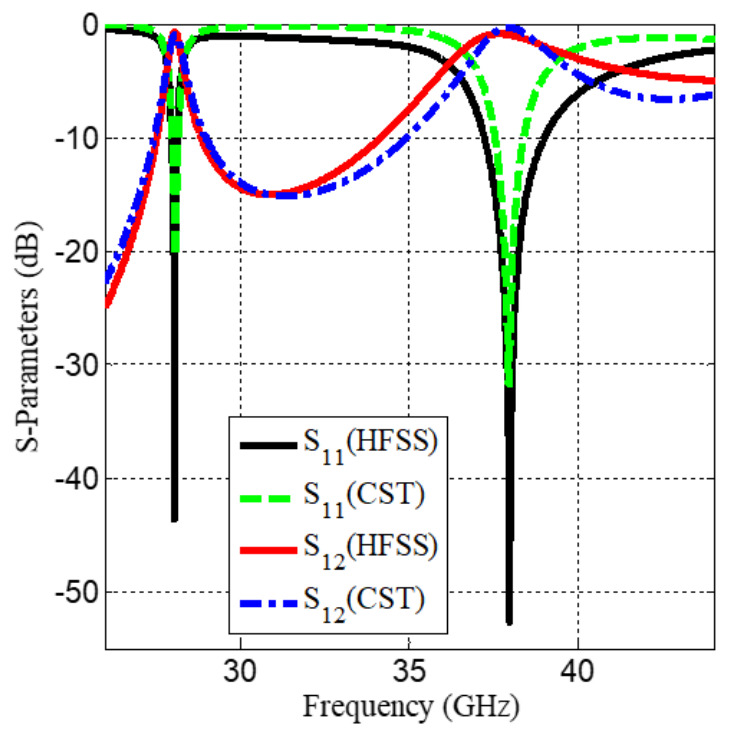
S -parameters simulated with HFSS and CST.

The final S-parameters can be extracted and plotted as shown in Figure 8, which represents the variation of the insertion loss and the return loss as a function of frequency based on the numerical parameters of Figure 3. It is clearly noted that this device is characterized by two passbands: the first one around 28 GHz, which is a narrow band with high selectivity, and another one around 38 GHz, which is a wide band.

According to the HFSS simulation results, the first bandwidth ranges from 27.88 GHz to 28.26 GHz at −3dB, with a passband width of 380 MHz. The minimum insertion loss (IL) reaches 0.67 dB, and the maximum return loss (RL) is 43.63 dB at the first resonant frequency of 28 GHz. In addition, for the second band centered on the resonance frequency of 38 GHz, the bandwidth extends from 36.35 GHz to 40 GHz at −3dB, giving a width of 3650 MHz. The IL reaches its minimum value of 0.86 dB at 37.58 GHz and 0.96 dB at the resonant frequency of 38 GHz, where a high RL of 52.65 dB is observed at the exact frequency of 37.96 GHz.

For the CST simulation, $I_{L}\approx 0.93\;\mathrm{dB}$, $R_{L}\approx 20\;\mathrm{dB}$ at 28 GHz, and $I_{L}\approx 0.31\;\mathrm{dB}$, $R_{L}\approx 31.7\;\mathrm{dB}$ at 38 GHz. Therefore, the fractional bandwidth Δ (*FBW*) and the quality factor *Q* are defined as follows [25]:

(8) $$FBW=\Delta =\left(\frac{f_{c2}-f_{c1}}{f_{c}}\right)\times 100\%,\;\mathrm{or}\;Q=\frac{f_{c}}{f_{c2}-f_{c1}}$$

where $f_{c1}$ and $f_{c2}$ are the first and second −3 dB cutoff frequencies, such that $f_{c}$ is the centered one. Therefore,

$$\Delta_{1}=1.35\%,\;Q_{1}=74.07,\;\Delta_{2}=9.56\%,\;Q_{2}=10.46.$$

**Figure 9:**
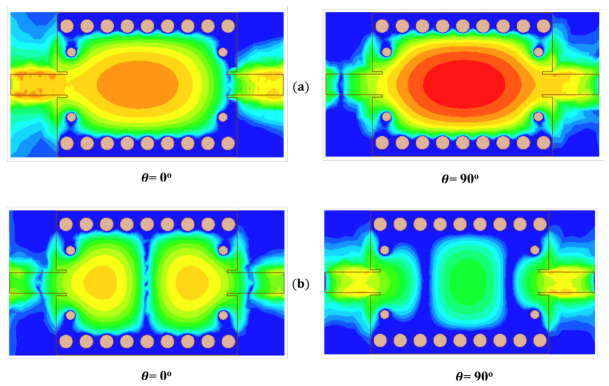
Electric field distribution at (**a**) $f_{TE_{101}}=28$ GHz; (**b**) $f_{TE_{201}}=38$ GHz.

According to Figure 9, which shows the electric field distribution on the SIW filter, we observe that the first resonance occurs at the ${\mathrm{TE}}_{101}$ mode, with a polarization phase θ equal to $\frac{\pi }{2}$ rad, at the desired frequency of 28 GHz. On the other hand, the second resonance corresponds to the ${\mathrm{TE}}_{201}$ mode, without phase shift, at the desired frequency of 38 GHz. It can be concluded that there is a 90-degree phase shift between the dominant modes $T_{E_{101}}$ and $T_{E_{201}}$, corresponding to the resonance frequencies 28 and 38 GHz, respectively, whose electric field intensity takes its maximum value of the order of $1.2\times 10^{5}$ (V/m) in the middle of the filter at the 28 GHz resonant frequency, of the $T_{E_{101}}$ mode with a phase θ equal to $\frac{\pi }{2}$, as shown in Figure 9a, where it equals the minimum values of the order of $3\times 10^{4}$ (V/m) for the same phase at the 38 GHz frequency of the $T_{E_{201}}$ mode.

## 4. Equivalent Circuit Model

**Figure 10:**
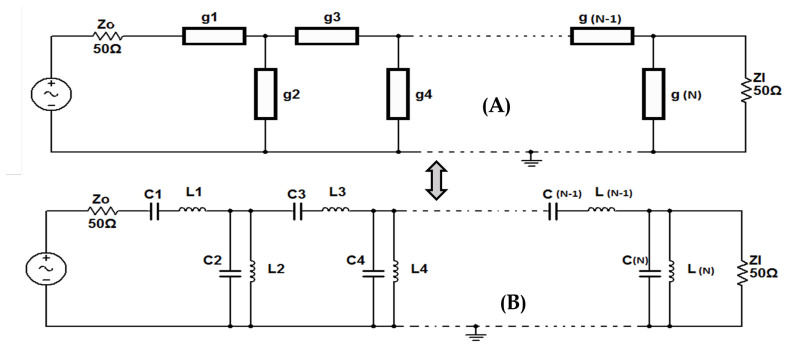
General equivalent circuit transformer: (**A**) normalized LPF elements; (**B**) electrical PBF components.

The proposed filter is introduced and investigated by the lumped-equivalent circuit (LEC) method, which is adopted to analyze the characteristics of the dual-band response based upon the low-frequency LEC [10]. This filter, designed using theoretical and analytical methods, can be considered equivalent to an electric circuit of Butterworth or Maximal flat type, based on the normalized low-passband filter (LPF) elements method “$g_{k}$”, referring to Kuroda’ transformer, as shown in Figure 10, where in our case, and in referring to the response of filter from the S-parameters shown in Figure 8, it is clearly noted that this filter reacts as a Butterworth or maximally flat filter of order N with dual bandwidth [2,4,26].

Generally, the $g_{k}$ elements can be calculated from

(9) $$g_{k}=2\sin\left(\frac{(2k-1)\pi }{2N}\right)\;\mathrm{for}\;k=1\;\mathrm{to}\;N.$$

**Figure 11:**
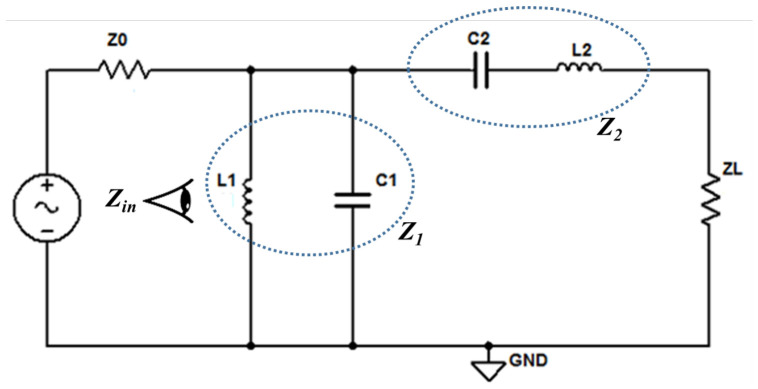
Second-order Butterworth PBF, first and second band, equivalent circuit.

And *N* is the filter’s order, which calculated as

(10) $$N=\frac{\log\left(10^{\frac{A}{10}}-1\right)}{2\log\left(\frac{f}{f_{c}}\right)}$$

where *A* is the insertion loss (IL) at the centered frequency $f_{c}$ within the bandwidth, and *f* is the lower first cutoff frequency. To transform the circuit as shown in Figure 11 from (A) to (B), the following relationships must be used:

(11) $$\left\{\begin{array}{cc} L_{i}\;\mathrm{in}\;\mathrm{series}\;(-),\;\mathrm{as}\;\mathrm{well}\;\mathrm{as}:\;L_{1},L_{3},\ldots ,L_{N-1}; & so,L_{i}=\frac{g_{i}Z_{0}}{\omega_{c}\Delta }\\ C_{i}\;\mathrm{in}\;\mathrm{series}\;(-),\;\mathrm{as}\;\mathrm{well}\;\mathrm{as}\;C_{1},C_{3},\ldots ,C_{N-1}; & so,C_{i}=\frac{\Delta }{g_{i}Z_{0}\omega_{c}} \end{array}\right.$$

(12) $$\left\{\begin{array}{cc} L_{j}\;\mathrm{in}\;\mathrm{parallel}\;(\|),\;\mathrm{as}\;\mathrm{well}\;\mathrm{as}\;L_{2},L_{4},\ldots ,L_{N}; & so,L_{j}=\frac{\Delta Z_{0}}{w_{c}g_{j}}\\ C_{j}\;\mathrm{in}\;\mathrm{parallel}\;(\|),\;\mathrm{as}\;\mathrm{well}\;\mathrm{as}\;C_{2},C_{4},\ldots ,C_{N}; & so,C_{j}=\frac{g_{j}}{\Delta Z_{0}w_{c}} \end{array}\right.$$

According to the previous formulas, it is important to note that they cannot be applied solely to single-band filters when determining equivalent circuits. In our case, the filter must be divided into two sections, each corresponding to one of the two bands. The first section will be centered around the 28 GHz frequency, while the second will focus on the 38 GHz frequency. Subsequently, we couple the two circuits to obtain the equivalent circuit for a dual-band filter. In addition, without delving into the complex calculations associated with Equation (9), we can conclude that the filter is of second order, as it includes two pairs of resonators, making it a basic filter configuration.

(13) $$N=2\;\Rightarrow \;k=1\;\mathrm{to}\;2,\;\mathrm{so}\;\mathrm{from}\;\mathrm{Equation}\;(9)\;\left\{\begin{array}{c} g_{1}=2,\\ g_{2}=1.414, \end{array}\right.\;\mathrm{and}\;g_{0}=g_{3}=1.$$

### 4.1. First and Second Bandwidth Separately (Around 28 GHz and 38 GHz)

**Figure 12:**
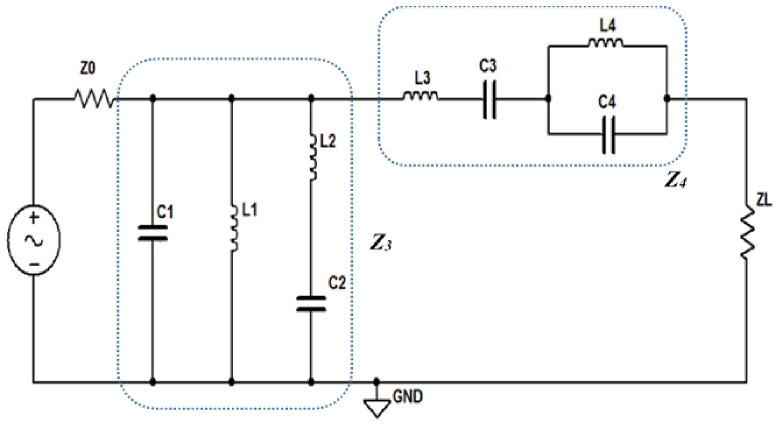
Second-order dual-band passbandButterworth filter.

As mentioned above, and in order to determine the equivalent circuit of the proposed dual-band filter, as well as to be able to apply the previous equations, especially Equations (9)–(13), it is necessary to first find the equivalent circuit of each separate band, as shown in Figure 11, because the SIW cavity can be described by an RLC equivalent resonant circuit [27]. Then, it will be necessary to carry out the assembly and coupling of the circuits in order to determine the equivalent circuit of the final filter, in accordance with Figure 12.

**Figure 13:**
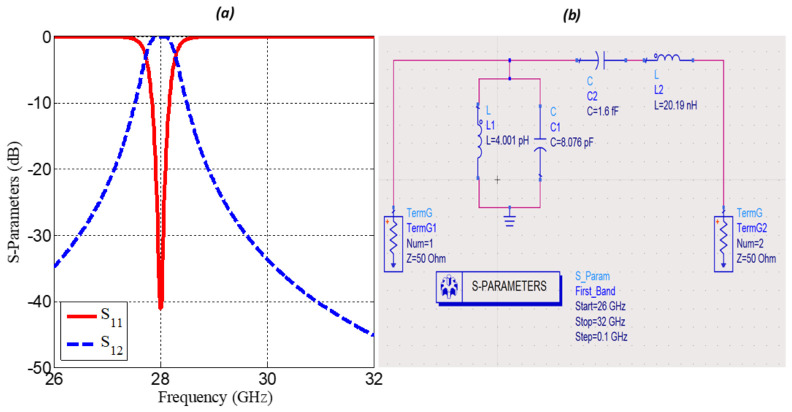
First band simulated with ADS. (**a**) S-parameters. (**b**) Equivalent circuit’s schematic.

**Figure 14:**
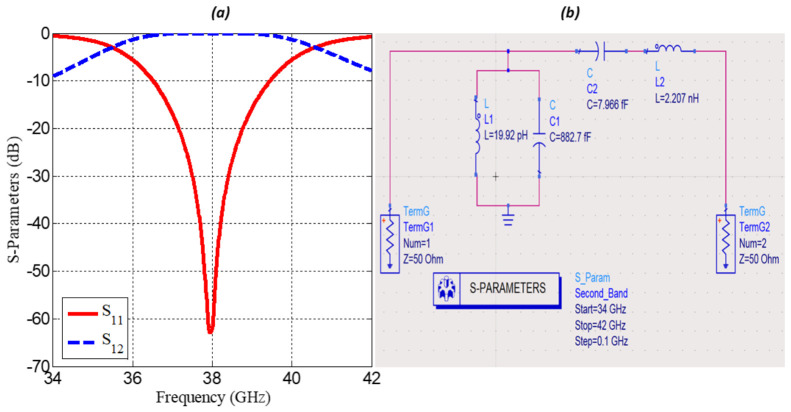
Second band simulated with ADS. (**a**) S-parameters. (**b**) Equivalent circuit’s schematic.

These two circuits were realized and simulated using Advanced Design System (ADS 2019) software; however, the value of each element for the first bandwidth is given in Table 2, and for the second one is given in Table 3, where the responses, represented as S-parameters, are plotted and extracted from the same software, as shown in Figure 13 and Figure 14, respectively.

The resonance frequency of electronic filters can be represented by the following general formula:

(14) $$f_{r}=\frac{1}{2\pi \sqrt{L\cdot C}}$$

We can approximate as that the resonance is dominated by the parallel branch $L_{1}\|C_{1}$ and adjusted by the series section $C_{2}$-$L_{2}$, as clearly observed in Figure 11 and Figure 12.

Therefore, for the parallel branch $L_{1}\|C_{1}$,

(15) $$\left\{\begin{array}{cc} \mathrm{first}\;\mathrm{band} & f_{r_{11}}=\frac{1}{2\pi \sqrt{\left(4.001\times 10^{-12}\right)\left(8.076\times 10^{-12}\right)}}\approx 27.9987\;\mathrm{GHz}\\ \mathrm{second}\;\mathrm{band} & f_{r_{12}}=\frac{1}{2\pi \sqrt{\left(19.92\times 10^{-12}\right)\left(882.7\times 10^{-15}\right)}}\approx 37.9549\;\mathrm{GHz} \end{array}\right.$$

And for the the series section $C_{2}$-$L_{2}$,

(16) $$\left\{\begin{array}{cc} \mathrm{first}\;\mathrm{band} & f_{r_{21}}=\frac{1}{2\pi \sqrt{\left(20.19\times 10^{-9}\right)\left(1.6\times 10^{-15}\right)}}\approx 28.0022\;\mathrm{GHz}\\ \mathrm{second}\;\mathrm{band} & f_{r_{22}}=\frac{1}{2\pi \sqrt{\left(2.207\times 10^{-9}\right)\left(7.966\times 10^{-15}\right)}}\approx 37.9576\;\mathrm{GHz} \end{array}\right.$$

We can approximate also that the effective resonant frequency is the one where the total impedance seen by the source $Z_{in}$ is minimal, or where the phase of the denominator passes through zero (capacitive/inductive regime change). Here,

$$Z_{in}=\left(Z_{L}+Z_{2}\right)\|Z_{1}$$

with

$$Z_{1}=L_{1}\|C_{1}=\frac{jL_{1}\omega }{1-C_{1}L_{1}\omega^{2}}$$

and

$$Z_{2}=C_{2}-L_{2}=-\frac{1}{jC_{2}\omega }+jL_{2}\omega$$

or

ω=2πf

so

$$Z_{in}=\left(R-\frac{1}{jC_{2}\omega }+jL_{2}\omega \right)\|\left(\frac{jL_{1}\omega }{1-C_{1}L_{1}\omega^{2}}\right)$$

with $Z_{L}=R=50$ Ω.

### 4.2. Equivalent Circuit of the Proposed Filter

As mentioned before, it is not possible to directly apply the previous equations and the $g_{k}$ elements method, as we have a dual bandwidth filter with two resonant frequencies $f_{1}$ and $f_{2}$, as well as two fractional bandwidths $\Delta_{1}$ and $\Delta_{2}$. For this, we rely on the circuit shown in Figure 11 in order to realize the final equivalent circuit, as illustrated in Figure 12.

The analysis is the same as the previous circuit, so the impedances $Z_{3}$ and $Z_{4}$ are responsible for achieving the resonant frequencies, 28 and 38 GHz, in the same design; $Z_{in}$ can be determined as follows:

$$Z_{in}=\left(Z_{L}+Z_{4}\right)\|Z_{3}$$

with

$$Z_{3}=[C_{1}\|L_{1}\left\|\left(L_{2}-C_{2}\right)\right],\;Z_{4}=\left[L_{3}-C_{3}-\left(L_{4}\|C_{4}\right)\right]$$

**Figure 15:**
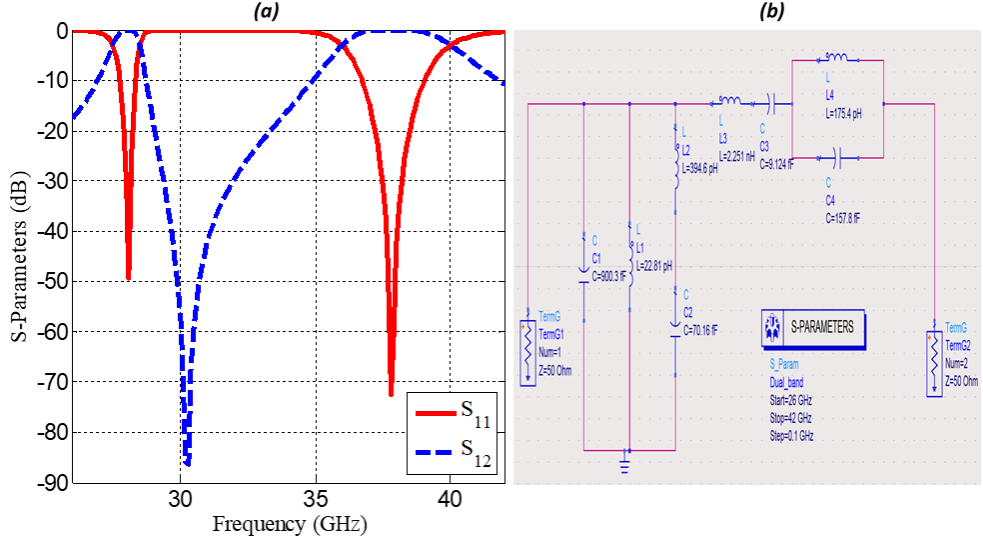
Second-order dual-band passband Butterworth filter’s simulated with ADS. (**a**) S-parameters. (**b**) Equivalent circuit’s schematic.

The resonant frequency corresponds to when $\mathrm{Img}(Z_{in})=0$. As the theoretical calculations are quite complex, we opted to simulate and optimize the circuit using Advanced Design System (ADS). The component values used for the simulation are listed in the accompanying Table 4, and the resulting response is illustrated in Figure 15, as generated by ADS. In Figure 12, the components $\left(L_{2}-C_{2}\right)$ and $\left(L_{4}\|C_{4}\right)$ act as regulators or disruptors within the circuit, influencing the resonant frequencies while also facilitating coupling by forming a bandpass filter. This configuration enables the creation of a circuit with two distinct bandwidths. As previously discussed, we combined two earlier circuits and introduced coupling through branch 4. This configuration inherently generated a transmission zero (TZ) at around 30.3 GHz, thereby forming a band-stop filter that returns the signal to zero at this frequency, as shown in Figure 15. To accurately identify the resonant frequencies, it is recommended to compute the transfer function of the overall circuit and determine the frequencies at which its argument becomes zero. An effective method to determine the resonant frequencies is through the calculation of the circuit’s transfer function, H(jω). By analyzing this function, one can derive the system’s poles and zeros, which directly correspond to the resonant frequencies. The transfer function, typically expressed as the ratio of output to input voltage (or current), is

(17) $$H\left(j\omega \right)=\frac{V{\left(j\omega \right)}_{\mathrm{out}}}{V{\left(j\omega \right)}_{\mathrm{in}}}.$$

It serves as a fundamental tool in characterizing the frequency response of the circuit, where the amplitude-squared transfer function for Butterworth filters that have an insertion loss IL ≈ 3 dB at the cutoff frequency $f_{c}$, is given by

(18) $$|S_{12}{\left(j\omega \right)|}^{2}=\frac{1}{1+{\left(\frac{\omega }{\omega_{c}}\right)}^{2N}}$$

where *N* is the degree or the order of filter, which corresponds to the number of reactive elements required in the lowpass prototype filter [2,4]. The resonant frequency corresponds to the value of ω for which |H(jω)| is maximum, so

(19) $$\frac{d}{d\omega }\left|H\left(j\omega \right)\right|=0\Rightarrow \omega =\omega_{r},$$

or

(20) $$\omega_{r}=2\pi f_{r},$$

and $f_{r}$ is the resonant frequency.

To assess the coherence between the S-parameter simulations obtained using HFSS and CST (as shown in Figure 8) and those derived from the equivalent circuit modeled in ADS (illustrated in Figure 15), we compare key filter parameters such as the resonant frequencies, insertion loss (transmission coefficient S12), return loss (or reflection coefficient S11), and the bandwidth of the passband. A strong correlation is observed between the results predicted by the equivalent circuit model in ADS and those obtained from full-wave electromagnetic (EM) simulations. While the overall agreement is substantial, minor discrepancies are noticeable. For example, the bandwidth of the first bandpass filter appears slightly wider in the ADS simulation compared to the EM simulations. Furthermore, RL and IL exhibit ideal behavior at the resonant frequencies, as summarized in Table 5. Classical circuit theories, such as Ohm’s and Kirchhoff’s laws, are valid for low-frequency applications where circuit dimensions are much smaller than the operating wavelength. But in high-frequency domains, where wavelengths can be on the order of a few millimeters and become comparable to or smaller than the circuit dimensions, even a slight variation in the circuit geometry (ΔL) can significantly affect its behavior due to the presence of distributed effects.

## 5. Comparative Study

The performance of the proposed dual-band passband filter is compared with other reported filters in Table 6. The developed filter is compact and has a good −3 dB FBW compared to most of the works. This work targets high-frequency mmWave (28–38 GHz), whereas all other references operate below 16 GHz except [5,8], which resonate in the Ka band. The proposed filter achieves exceptional return loss (RL) (43–52 dB), the best among all, and its insertion loss (IL) is moderate (0.7–0.86 dB), and better than most, except [5,11,19], which are extremely low. Otherwise, the FBW is modest (1.36–9.61%), and not as wide as [11,12,13,21] which are designed with different techniques, as mentioned in Table 6. However, this is expected for higher-frequency SIW designs. As for size, it is relatively compact for the operation of mmWave, and of a very small size compared to others (6.23 mm × 10.81 mm). As represented in Table 6, the size is at the guided wavelength at a lower cutoff frequency $\lambda_{g}$, so we cannot know the exact size in mm; on the other hand, [19] is also compact, but for much lower frequencies.

Therefore, this work presents a high-performance mmWave bandpass filter with superior return loss, competitive insertion loss, reasonable compactness, and designed using SIW, which supports high-frequency integration.

## 6. Conclusions

In this work, a dual-band filter is proposed based on theoretical analysis and electromagnetic simulation results only, without physical fabrication or experimental validation, and designed without incorporating reconfigurability, which typically requires sensitive components, such as PIN diodes, that operate in the millimeter-wave range to achieve high frequencies in the tens of gigahertz range. The first band is a narrow band around 28 GHz, while the second one is a wider band around 38 GHz, both of which are designated for 5G applications. The device is designed using substrate integrated waveguide (SIW) technology, based on an inductive shunt iris with high performance, with a very high return loss (RL), having values of 40 dB and 50 dB at 28 GHz and 38 GHz, respectively.

The filter has a simple and compact geometric design, with overall dimensions of 10.81 mm in length and 6.23 mm in width (($L_{\mathrm{tot}}$ × $W_{\mathrm{tot}}$) ≈ (3/2 × 1)$\lambda_{g}$/2), making it small and efficient. It has a planar structure with a thickness of 0.508 mm. Furthermore, it exhibits high selectivity at 28 GHz, characterized by a narrow band with a fractional bandwidth of −3 dB (FBW) of approximately 1.36%, and another wider band with FBW of approximately 9.61%. Consequently, the filter operates within the frequency ranges of approximately 27.84 GHz to 28.44 GHz and 34.91 GHz to 44.66 GHz.

Due to the existence of a simplified equivalent circuit for this filter, its design and fabrication demonstrate its feasibility for practical fabrication. Additionally, its compact size—with an overall length of 10.81 mm and a total width of approximately 6.23 mm—enhances its performance. The very small via diameter of 0.55 mm, the resonator diameter of 0.36 mm, and the thin substrate thickness of just 0.508 mm classify it as an extra-planar component. Furthermore, it exhibits dual resonance at two distinct frequencies—28 GHz and 38 GHz—using the same structure without requiring any modifications. These features make it an excellent candidate to serve as a key component in circuits and electronic devices operating in the millimeter-wave range and for next-generation communication systems and related technologies, especially in various 5G (fifth-generation) applications.

## Figures and Tables

**Table 1:** Filter parameters.

Parameter	Value (mm)	Parameter	Value (mm)
$L_{\mathrm{sub}}$	10.81	*d*	0.55
$W_{\mathrm{sub}}$	6.23	$d_{1}$	0.36
$L_{\mathrm{siw}}$	6.40	*p*	0.80
$W_{\mathrm{siw}}$	4.65	$k_{i}$	2.58
*h*	0.508	$l_{i}$	6
$w_{1}$	0.825	$l_{k}$	1.84
*t*	0.017	*e*	0.10
$l_{s}$	0.40	$\lambda_{c}$	8.40

**Table 2:** Component values of the first bandwidth.

Parameter	Value
$Z_{0}$	50 Ω
$Z_{L}$	50 Ω
$C_{1}$	8.076 pF
$L_{1}$	4.001 pH
$C_{2}$	1.6 fF
$L_{2}$	20.19 nH

**Table 3:** Component values of the second bandwidth.

Parameter	Value
$Z_{0}$	50 Ω
$Z_{L}$	50 Ω
$C_{1}$	882.7 fF
$L_{1}$	19.92 pH
$C_{2}$	7.966 fF
$L_{2}$	2.207 nH

**Table 4:** Dual-band filter’s circuit component values.

Parameter	Value
$C_{1}$	900.3 fF
$L_{1}$	22.81 pH
$C_{2}$	70.16 fF
$L_{2}$	394.6 pH
$C_{3}$	9.124 fF
$L_{3}$	2.251 nH
$C_{4}$	157.8 fF
$L_{4}$	175.4 pH
$Z_{0}$	50 Ω
$Z_{L}$	50 Ω

**Table 5:** Comparison between the simulation results using HFSS, CST, and those of equivalent circuit using ADS.

Mod.	No. of Bands	$f_{r}$ (GHz)	BW (−3 dB) (MHz)	$S_{11}$ at [$f_{r}$] (dB)	$S_{12}$ at [$f_{r}$] (dB)
HFSS	2	28.05	380	−43.63	−0.67
		37.96	3650	−52.62	−0.94
CST	2	28.07	425.6	−20.30	−0.93
		37.95	2454.8	−31.76	−0.28
ADS	2	28.10	1000	−49.34	−5.05 × 10^−5^
		37.80	4000	−72.39	−2.50 × 10^−7^

**Table 6:** Performance comparison of reported bandpass filters.

Ref.	Technology	$f_{r}$ (GHz)	FBW (%)	RL (dB)	IL (dB)	Size ($\lambda_{g}^{2}$)	Sim./ Meas.
[5]	SIW	28.26	3.63	>25	0.04	0.99	Sim.
[7]	CSMDMSIW	10.01	3.98	>20	1.52	4.84	Meas.
		10.03	3.94		1.84		
[8]	SIW	26.87	19.50	>10.41	2.47	5.44	Meas.
[9]	HMSIW with TZs	10.00	5.30	>20	2.40	1.75	Meas.
[11]	D-SCLR	7.40	32.40	>17	0.23	0.39	Meas.
[12]	MMRs	15.40	109	20	0.40	0.49	Meas.
[13]	CSSIW	13.00	48	14	1.10	1.53	Meas.
[14]	PSIWRC	10.00	3.30	>30	1.55	2.00	Meas.
[18]	TLSSIW using NRN	10.00	8.09	>20	2.00	1.12	Meas.
[21]	USSIW	8.5	42	>11	<1.10	0.78	Meas.
**This work**	**SIW**	**28.05** **37.96**	**1.36** **9.61**	**43.63** **52.65**	**0.68** **0.86**	**0.33**	**Sim.**

## Data Availability

The original contributions presented in this study are included in the article. Further inquiries can be directed to the corresponding authors.

## Abbreviations

The following abbreviations are used in this manuscript:

RF	Radio frequency
$f_{r}$	Frequency of resonance
FBW	Fractional bandwidth
TZs	Transmission zeroes
SIW	Substrate integrated waveguide
CSMDMSIW	Combines single-mode and dual-mode substrate-integrated waveguides
WGCW/SIW	Wideband grounded coplanar waveguide to SIW transition
HMSIW	Half-mode substrate-integrated waveguide
D-SCLR	Diamond-shaped coupled line resonator
MMRs	Multi-mode resonators
CSSIW	Comb-slotted substrate integrated waveguide
PSIWRC	Perturbed SIW rectangular cavities
TLSSIW	Tri-layer stacked SIW
NRN	Non-resonant nodes
USSIW	U-slotted substrate-integrated waveguide
Sim.	Simulated
Meas.	Measured
